# Employee perspectives on safeguarding information: An exploratory study in a multinational energy corporation

**DOI:** 10.1371/journal.pone.0349656

**Published:** 2026-10-07

**Authors:** Mohamed Mohiya

**Affiliations:** Human Resources Management Department, College of Business, King Khalid University, Abha, Saudi Arabia; Comenius University in Bratislava: Univerzita Komenskeho v Bratislave, SLOVAKIA

## Abstract

Information security has become a critical organizational and HR priority in an era of escalating cyber threats. Despite extensive technological investments in cybersecurity infrastructure, the human dimension of information safeguarding remains underexplored, creating significant vulnerabilities in organizational defense mechanisms. This exploratory research investigates employees’ reactions, thoughts, and experiences regarding information safeguarding practices within a large multinational energy corporation in Saudi Arabia. Grounded in Protection Motivation Theory (PMT), this study examines how employees perceive threats to organizational information assets and their behavioral responses to safeguarding imperatives. The research employs a unique dual-method qualitative approach, analyzing 87 firsthand computerized employee comments — one comment contributed by each of 87 distinct employees — extracted from an internal electronic system and conducting 24 semi-structured interviews with full-time employees across various organizational levels. Through systematic thematic analysis, the study identifies five major themes: perceived vulnerability and threat awareness, individual responsibility and accountability, organizational security culture, technical versus human factors in information protection, and knowledge gaps and training. Findings reveal a persistent paradox: heightened threat awareness does not automatically translate into consistent protective behaviors, a pattern moderated by organizational culture, variations in self-efficacy, and response-cost barriers. The study extends PMT to an underexplored context of critical infrastructure. It offers actionable insights for practitioners seeking to cultivate security cultures, address self-efficacy gaps, and design human-centered security interventions in high-stakes organizational environments.

## 1. Introduction

The contemporary organizational landscape is characterized by unprecedented dependence on digital information systems, rendering information assets among the most valuable and vulnerable organizational resources [1]. Within the global energy sector, information security transcends conventional business concerns to encompass national security imperatives, as energy infrastructure constitutes critical national assets whose compromise could precipitate economic disruption and geopolitical instability. The digitalization of energy operations has amplified both operational efficiency and security vulnerabilities, creating complex challenges for organizations navigating the tension between accessibility and the protection of sensitive information.

Saudi Arabia’s energy sector occupies a pivotal position in the global economy, with the Kingdom serving as the world’s leading petroleum exporter and holding approximately 17% of global proven oil reserves [2]. The sector’s strategic importance has intensified efforts to safeguard proprietary exploration data, production technologies, and operational intelligence from increasingly sophisticated cyber adversaries. Recent years have witnessed escalating cyber threats targeting energy infrastructure globally, with state-sponsored actors and criminal enterprises seeking to compromise sensitive information for economic, political, or strategic advantage [3,4]. These evolving threats necessitate comprehensive security frameworks that integrate technological defenses with robust human-centered practices.

Despite substantial investments in cybersecurity technologies, including firewalls, intrusion detection systems, and encryption protocols, organizations continue to experience security breaches attributable to human factors. Research consistently identifies employees as both the weakest link and potentially the strongest defense in organizational security architectures [5,6]. However, scholarly attention has predominantly focused on technical security mechanisms, with comparatively limited exploration of employee perspectives, experiences, and behaviors regarding information safeguarding. This empirical gap is particularly pronounced in the energy sector and in Middle Eastern organizational contexts, where cultural factors may shape security attitudes and practices in distinctive ways. It is worth noting that a body of information security research has been conducted within Saudi Arabian and broader Middle Eastern institutional settings; however, much of this work has achieved limited international visibility in English-language scholarship [7,8], creating an apparent — though partial — gap in the accessible literature.

### 1.1. Research motivation, problem statement, and research gaps

The motivation for this research emerges from the confluence of several critical factors. First, the increasing frequency and sophistication of cyberattacks targeting energy infrastructure demand a deeper understanding of organizational vulnerabilities beyond technical systems. Second, the persistent gap between organizational security policies and employee compliance behaviors suggests fundamental disconnects in how security imperatives are communicated, understood, and enacted [9,10]. Third, the scarcity of qualitative research that captures authentic employee voices on information security limits organizations’ capacity to design human-centered security interventions [11]. Fourth, the unique sociocultural context of Saudi Arabian organizations remains underexplored in international information security scholarship, despite the Kingdom’s critical role in global energy markets [7,8].

The central research problem concerns the limited understanding of how employees within a large multinational energy corporation perceive, experience, and respond to organizational information-safeguarding imperatives. While organizations implement comprehensive security frameworks that encompass technical controls, policies, and training programs, there is limited insight into employees’ actual thought processes, behavioral motivations, and experiential realities regarding information protection. This knowledge deficit constrains organizational capacity to cultivate robust security cultures and to develop interventions that resonate with employees’ lived experiences and contextual realities.

Analysis of the existing literature reveals several critical gaps that warrant investigation. Theoretically, information security research has insufficiently leveraged behavioral psychology theories to explain employee security behaviors, with limited application of Protection Motivation Theory specifically to organizational information safeguarding contexts within the energy sector [12,13]. Methodologically, the field exhibits an overreliance on quantitative surveys and experimental designs, while qualitative approaches that capture rich employee narratives remain underutilized [12,14]. Empirically, scholarship lacks in-depth exploration of employee perspectives through authentic organizational data sources such as naturally occurring employee comments and extended interviews [11]. Contextually, research disproportionately focuses on Western organizational settings, with Middle Eastern contexts, particularly Saudi Arabian organizations, receiving minimal scholarly attention [8,15]. Sectorally, while critical infrastructure protection garners policy attention, micro-level employee experiences within energy-sector organizations remain underdocumented [3,16].

### 1.2. Theoretical foundation

This research adopts Protection Motivation Theory (PMT) as its theoretical foundation to illuminate how employees cognitively appraise information security threats and formulate protective behavioral responses. Originally developed to explain health-protective behaviors, PMT has demonstrated robust explanatory power across diverse domains, including information security [13,17,18]. The theory posits that individuals engage in protective behaviors through two cognitive appraisal processes: threat appraisal, encompassing perceived severity and vulnerability, and coping appraisal, encompassing response efficacy, self-efficacy, and response costs [18]. In organizational information security contexts, PMT suggests that employees’ safeguarding behaviors result from evaluating both the seriousness of potential security breaches and their confidence in their ability to execute protective measures effectively [13,19].

PMT provides particularly appropriate theoretical grounding for this exploratory research for several reasons. First, it acknowledges the cognitive complexity underlying security behaviors, moving beyond simplistic compliance models. Second, it accommodates contextual factors that influence threat and coping appraisals, enabling the investigation of organizational and cultural dynamics. Third, it offers actionable dimensions for organizational intervention, identifying leverage points for enhancing employee security behaviors. Fourth, its application to organizational information security within the energy sector context remains underexplored, presenting opportunities for theoretical extension. By employing PMT as an analytical lens, this research examines how employees’ threat perceptions, vulnerability assessments, and coping capabilities shape their information-safeguarding practices, thereby contributing to theoretical advancement and generating practical insights.

### 1.3. Research aim, objectives, and questions

*Research Aim:* This research aims to explore and understand employees’ perspectives, experiences, and behaviors regarding information safeguarding practices within a large multinational energy corporation in Saudi Arabia, using Protection Motivation Theory to illuminate the cognitive and contextual factors that influence employees’ security behavior.

*Research Objectives:*

1. To identify and analyze employees’ perceptions of information security threats and their perceived vulnerability to such threats within the organizational context.
2. To examine employees’ understanding of their individual responsibilities and the organizational expectations regarding information protection behaviors.
3. To explore the coping mechanisms, self-efficacy beliefs, and behavioral responses employees employ when confronted with information security challenges.
4. To investigate contextual factors within the organizational culture and the Saudi Arabian setting that facilitate or constrain effective information safeguarding practices.

*Central Research Question:* How do employees at a multinational energy corporation in Saudi Arabia perceive, experience, and respond to organizational information-safeguarding imperatives, and what factors shape their protective behaviors?

*Exploratory Research Questions:*

**ERQ1:** How might threat appraisal — encompassing perceived severity and vulnerability — manifest in employees’ accounts of information security within this organizational context?

**ERQ2:** In what ways do self-efficacy beliefs and perceived response efficacy appear to shape employees’ information protection behaviors within this context, consistent with PMT’s coping appraisal dimension?

**ERQ3:** How might organizational culture and management commitment facilitate or constrain the translation of security awareness into actual protective behaviors in this setting?

**ERQ4:** What contextual factors specific to the Saudi Arabian energy sector — including national security considerations and prior cyber incident experience — appear to shape employee perspectives on information safeguarding?

### 1.4. Methodological novelty

This research employs an innovative dual-source qualitative methodology, distinguishing it from conventional information security studies. First, the analysis of 87 firsthand, computerized employee comments — one comment per employee, contributed by 87 distinct employees — extracted from an internal electronic system provides unfiltered, spontaneous employee perspectives captured in their natural organizational context rather than through researcher-prompted responses. This documentary data represents authentic employee voices expressing genuine concerns, experiences, and suggestions regarding information security, offering ecological validity often absent in structured survey research [12]. It is important to acknowledge that these comments were solicited through an internal communication initiative following a significant cyber incident, rather than arising as wholly spontaneous, naturally occurring organizational discourse; this contextual feature is treated as an analytically relevant characteristic of the dataset. Second, 24 semi-structured interviews enable deeper exploration of themes that emerge from documentary analysis while accommodating participants’ elaboration on complex experiences. This methodological triangulation enhances the credibility and comprehensiveness of findings while capturing both the breadth and depth of employee perspectives [20,21]. The combination of naturalistic documentary data and purposive interview data represents a methodologically rigorous and innovative approach to understanding employees’ experiences with information security.

### 1.5. Saudi Arabian energy sector context

Saudi Arabia’s energy sector represents a cornerstone of the national economy, contributing approximately 50% of gross domestic product and 70% of export earnings [22]. The Kingdom’s Vision 2030 strategic framework [23] emphasizes economic diversification while maintaining energy sector competitiveness through technological innovation and operational excellence. This transformation necessitates extensive digitalization initiatives, while simultaneously expanding the cyber-attack surface and elevating information security imperatives. The sector’s strategic importance attracts persistent cyber threats from state and non-state actors seeking competitive intelligence, operational disruption, or geopolitical leverage. Understanding employee perspectives within this high-stakes context provides critical insights for safeguarding national strategic assets while illuminating how organizational culture, national security consciousness, and sector-specific pressures shape information security behaviors. Tentatively, the Saudi Arabian context may offer transferable insights to other nations whose critical energy infrastructure faces similar security challenges in an increasingly interconnected global landscape.

## 2. Literature review

### 2.1. Conceptualizing information security behavior in organizations

Information security encompasses the protection of the confidentiality, integrity, and availability of information assets through technical, administrative, and physical controls [1]. However, scholars increasingly recognize that technological sophistication alone cannot ensure organizational security, as human behavior constitutes both the greatest vulnerability and potential strength in security architectures [5,6]. Siponen and Vance [24] argue that information security must be understood as a sociotechnical phenomenon that requires the integration of technical systems, organizational processes, and human behavior. This conceptualization shifts attention from purely technical defenses to understanding why employees engage in or deviate from protective behaviors, emphasizing the need for behavioral theories that explain security decision-making. Organizational information security culture represents the collective values, beliefs, and behavioral norms regarding information protection shared among organizational members [25,26]. Research demonstrates that strong security cultures are associated with fewer security incidents and greater employee engagement in protective practices [27,28]. However, Karlsson et al. [29] found that employees often perceive security as an organizational rather than an individual responsibility, suggesting a disconnect between intended security cultures and employees’ role perceptions. These findings raise critical questions about the mechanisms by which organizational culture translates into individual behavioral change—questions that existing research addresses inadequately, relying on predominantly quantitative methodologies that cannot capture the nuanced meanings employees ascribe to security responsibilities.

### 2.2. Theoretical foundations: Protection motivation theory in information security

Protection Motivation Theory, originally developed by Rogers [17] to explain health-protective behaviors, posits that individuals engage in protective actions through cognitive appraisals of threats and coping capabilities. The theory identifies two parallel processes: threat appraisal, encompassing perceived severity and vulnerability, and coping appraisal, encompassing response efficacy, self-efficacy, and response costs [18]. Within information security contexts, Boss et al. [13] demonstrated that threat severity, vulnerability, and self-efficacy significantly predict employees’ security behavioral intentions, while Johnston and Warkentin [19] found that fear appeals based on PMT constructs enhance security compliance. However, Herath and Rao [9] noted that PMT alone provides incomplete explanations, as organizational sanctions and normative beliefs also substantially influence security behaviors.

Despite PMT’s growing application in information security research, significant theoretical gaps persist. Recent scholarship has further extended protection- and deterrence-based models in organizational security contexts: Cheng et al. [30] showed that social control and deterrence mechanisms jointly shape employees’ violations of security policy; Safa et al. [31] demonstrated that information security knowledge sharing, collaboration, intervention, and experience shape employees’ attitudes towards compliance; and Li et al. [32] showed that employees’ awareness of cybersecurity policies, together with their threat and coping appraisals, is associated with their cybersecurity behavior, underscoring the need for the kind of contextualized qualitative inquiry undertaken here. First, most PMT-based studies employ quantitative surveys that measure behavioral intentions rather than actual behaviors, thereby limiting understanding of intention-behavior gaps [12,14]. Second, PMT research inadequately addresses contextual factors that may moderate relationships between threat appraisals, coping appraisals, and behaviors—particularly organizational culture, management commitment, and peer influences [33,34]. Third, PMT applications remain concentrated in Western organizational contexts, with limited exploration of how cultural values shape threat perceptions and coping strategies [8,15]. Finally, PMT-based information security research rarely examines critical infrastructure sectors where security carries heightened consequences [3,35]. These theoretical gaps necessitate extending PMT through qualitative exploration of employees’ lived security experiences across diverse organizational and cultural contexts.

Research demonstrates that organizational culture significantly influences employee security behaviors beyond formal policy compliance. Da Veiga and Eloff [26] found that organizations with strong security cultures experience fewer security incidents and demonstrate higher employee engagement with protective practices. Employees within supportive security cultures perceive information protection as a shared responsibility rather than solely an IT department function. However, cultivating robust security cultures requires more than policy pronouncements; it demands authentic management commitment, consistent communication, visible modeling of security behaviors by leaders, and integration of security considerations into everyday workflows. Understanding how employees experience and interpret organizational security culture provides critical insights for culture transformation initiatives.

### 2.3. Relevant research work

The scholarly literature on employee information security behaviors has expanded substantially over the past decade, yet significant gaps persist. A critical evaluation of relevant research reveals both advances and limitations informing the present study.

*Quantitative Compliance Studies*

Substantial research has examined information security policy compliance through quantitative methodologies. Bulgurcu et al. [10] surveyed 464 employees across multiple organizations and found that information security awareness, attitude toward compliance, and perceived effectiveness of security policies significantly predicted compliance intentions. Their research advanced the understanding of compliance antecedents but remained limited to self-reported behavioral intentions rather than actual behaviors. Similarly, Herath and Rao [9] investigated deterrence theory and social influence factors affecting compliance with security policy through a survey of 297 employees. While demonstrating that both deterrent measures and social norms influence compliance, their study’s cross-sectional design precluded examination of behavioral change processes or contextual nuances.

Pahnila et al. [36] applied the technology acceptance model constructs to information security behavior, surveying employees about factors influencing the adoption of security technologies. Their quantitative approach yielded generalizable findings on perceived usefulness and ease of use as predictors of compliance. However, these studies share common limitations: reliance on survey methods that limit response options, a focus on behavioral intentions rather than enacted behaviors, and insufficient attention to contextual factors that shape security practices. Furthermore, quantitative approaches inadequately capture the complexity of employees’ lived experiences, reasoning processes, and contextual influences on security behaviors.

*Qualitative Explorations of Security Practices*

A smaller body of qualitative research has examined employee security behaviors through interpretive lenses. Beautement et al. [6] conducted ethnographic observations and interviews in a financial services organization, revealing that employees make pragmatic trade-offs between security compliance and work efficiency. Their research illuminated how formal security policies often conflict with operational realities, prompting employees to develop workarounds enabling task completion. This finding underscores the limitations of policy-centric approaches that neglect situated work practices.

Karlsson et al. [11] employed a grounded theory approach, conducting 18 interviews to examine how employees conceptualize information security responsibilities. Their analysis revealed that employees view security primarily as an organizational concern rather than an individual responsibility, suggesting a disconnect between organizational expectations and employees’ perceptions of their roles. While methodologically rigorous, their study focused exclusively on Scandinavian organizations, limiting transferability to other cultural contexts. Additionally, their reliance solely on interview data precluded triangulation with authentic organizational documents reflecting spontaneous employee perspectives.

*Energy Sector Security Research*

Research specifically addressing information security within energy sector organizations remains remarkably sparse. Cherdantseva and Hilton [3] reviewed information security research, noting that critical infrastructure sectors, including energy, receive disproportionately limited scholarly attention despite heightened security imperatives. Bronk and Tikk-Ringas [37] analyzed the 2012 Shamoon cyberattack on Saudi Aramco, documenting how the compromise of approximately 30,000 corporate workstations exposed the strategic vulnerability of Saudi Arabia’s energy infrastructure, but offering limited insight into employee behavioral dimensions. Their analysis emphasized strategic and policy responses, leaving the human factors that shape employees’ everyday protective behavior largely unexamined.

Knowles et al. [4] surveyed cybersecurity management in industrial control systems, which underpin energy operations, highlighting that effective security management in these environments depends on organizational processes, standards, and staff capabilities as well as on technical controls. Their review identified gaps in organizational preparedness but did not explore employees’ own perspectives regarding information safeguarding responsibilities and practices. Ani et al. [16] evaluated the cybersecurity capacity of the industrial workforce, demonstrating that gaps in employees’ security knowledge and skills constitute significant security risks. However, their quantitative approach could not illuminate the contextual factors, motivations, and experiential realities underlying risky behaviors. The energy sector literature thus reveals a critical gap: while acknowledging human vulnerabilities, research has not explored in depth employee perspectives, experiences, and behavioral motivations regarding information safeguarding in real-world energy sector organizational contexts.

*Middle Eastern and Saudi Arabian Context*

Information security research in Middle Eastern contexts, particularly within Saudi Arabian organizations, remains considerably underdeveloped. Alhogail [7] designed and validated an information security culture framework, showing that security culture is shaped by interacting human, organizational, and environmental dimensions. This framework-oriented study provided a useful foundation but could not explore the nuanced ways cultural factors shape employees’ security perceptions and behaviors. Alzahrani [8] empirically examined the factors that shape users’ compliance with information security policies, and AlGhamdi et al. [38] systematically reviewed information security governance challenges and critical success factors, identifying managerial, resource, and structural obstacles. However, this research focused on compliance determinants and organizational-level governance rather than on employee-level experiences and behaviors.

The cultural context of Saudi Arabian organizations presents unique considerations for information security research. The Kingdom’s collectivist cultural orientation emphasizes group harmony and hierarchical deference, which may influence how employees perceive individual versus collective security responsibilities. Additionally, the strategic importance of the energy sector to Saudi national development heightens security consciousness, which may shape employee attitudes in distinctive ways. Despite these unique contextual factors, the scholarly literature offers insufficient insight into how employees in the Saudi Arabian energy sector experience and enact information-safeguarding practices. This research addresses these critical contextual and sectoral gaps through an in-depth qualitative exploration of employee perspectives within an authentic Saudi Arabian energy-sector organizational setting.

*Synthesis and Research Positioning*

A critical evaluation of the existing literature reveals several patterns and gaps. First, quantitative compliance research dominates the field, providing generalizable insights into policy adherence but insufficiently capturing the complexity of employees’ security experiences. Second, qualitative research offers richer insights but remains limited in scope, cultural diversity, and sectoral representation. Third, energy sector research emphasizes technical vulnerabilities while underexploring human behavioral dimensions. Fourth, Middle Eastern and Saudi Arabian contexts remain dramatically underrepresented in international scholarship. Fifth, research rarely employs multiple qualitative data sources to comprehensively triangulate employee perspectives.

This research addresses these gaps through several distinctive contributions. It employs the Protection Motivation Theory to examine employee information safeguarding in an underexplored context, thereby extending its theoretical application. It utilizes an innovative dual-source qualitative methodology that combines authentic organizational documentary data with in-depth interviews, thereby enhancing methodological rigor and ecological validity. It focuses specifically on the critical energy sector, where information security carries national strategic implications. It illuminates the Saudi Arabian organizational context, contributing contextual knowledge to predominantly Western-centric scholarship. By addressing these theoretical, methodological, empirical, contextual, and sectoral gaps, this research advances scholarly understanding and generates actionable insights for practitioners managing information security in complex, high-stakes organizational environments.

## 3. Materials and methods

This research adopts an exploratory qualitative case study design to investigate employee perspectives on information safeguarding within a large multinational energy corporation operating in Saudi Arabia. The single case study approach enables in-depth exploration of a specific organizational context while yielding rich insights into complex behavioral and cultural phenomena. Yin [21] argues that case study methodology is particularly appropriate for investigating contemporary phenomena in real-life contexts where the boundaries between the phenomenon and its context may be unclear. Information safeguarding behaviors are deeply embedded in organizational contexts, making case study methodology ideally suited to capturing the complexity of employees’ experiences, perceptions, and practices.

### 3.1. Research setting and context

The research was conducted within a major multinational energy corporation headquartered in Saudi Arabia, representing one of the world’s largest integrated energy and chemicals companies. The organization employs over 70,000 employees globally, with operations spanning petroleum exploration and production, refining, distribution, and petrochemical manufacturing. The corporation’s information assets include proprietary exploration technologies, reservoir data, production processes, financial information, and strategic planning documents, all of which constitute high-value targets for cyber adversaries and competitive intelligence operations.

The organization experienced a significant cyberattack several years prior to data collection, giving employees firsthand experience of information security breaches and their consequences. This organizational history creates a particularly information-rich research context in which employees are highly aware of security vulnerabilities and organizational responses. The Saudi Arabian energy sector’s strategic national importance further amplifies security consciousness, as sector organizations are understood to face persistent cyber threats from state-sponsored actors and criminal enterprises. This unique confluence of factors makes the organization an exemplary case for exploring perspectives on safeguarding employee information.

### 3.2. Participants and sampling strategy

This research employs a purposive sampling strategy designed to capture diverse employee perspectives across organizational levels, functional areas, and tenure categories. Purposive sampling enables the deliberate selection of information-rich cases likely to yield insights that address research objectives [20]. The sampling strategy aimed to include employees with varying levels of technical expertise, security responsibilities, and exposure to organizational information assets.

For the documentary analysis component, 87 employee comments were extracted from an internal electronic system in which employees voluntarily shared their perspectives on organizational cybersecurity and information protection practices. These comments were solicited through an internal communication initiative encouraging employee input on safeguarding practices following a significant cyber incident experienced by the organization. To clarify the nature of this data source: the internal electronic system permitted only one submission per employee, with each submission authenticated against an individual employee account. The 87 comments therefore originate from 87 distinct employees, so that the comment count and the contributor count are identical and no employee is represented more than once in the documentary dataset. All comments were de-identified on export, so that no comment can be traced back to a named individual. The initiative was communicated to all employees within the relevant business units (approximately 350 employees), giving a participation rate of 24.9% (87 ÷ 350), reported hereafter as approximately 25%. This unique-contributor basis is applied consistently throughout the manuscript: the documentary component contributes 87 participants, and the interview component contributes 24 participants, for a total of 111 unique participants (Tables 1 and 4). It is important to note that these comments were solicited following a significant cyber incident, which may have elevated security consciousness above typical baseline levels; this contextual feature is treated as an analytically significant characteristic of the data rather than a limitation to be minimized. Due to the sensitive and confidential nature of the organizational data — reflecting real security incidents and internal operational practices — and the anonymity commitments made to participants and the host organization, the raw dataset cannot be deposited in a public repository. An anonymized illustrative codebook with representative coded excerpts has been prepared as S1 File.

For the interview component, 24 employees participated in semi-structured interviews lasting 45–60 minutes each. The sampling frame comprised employees across five functional departments (operations, engineering, IT/cybersecurity, administration, and finance) who had been employed for at least three months and had regular access to organizational information systems. Within this frame, purposive sampling was applied to deliberately select participants representing diverse organizational demographics, including management levels (senior managers, middle managers, supervisors, professional staff), functional areas, technical expertise levels (IT professionals, employees with moderate technical knowledge, end-users with limited technical training), and organizational tenure (less than 5 years, 5–15 years, over 15 years). Initial participant contacts within each department were identified through the HR liaison, who facilitated introductions and ensured that the researcher independently confirmed willingness to participate. 30 employees were approached; 24 agreed to participate, yielding a response rate of 80%. The sample size of 24 was guided by theoretical saturation: data collection continued until no substantially new codes or themes emerged across successive interviews, which was achieved by the 20th interview, with four additional interviews conducted to confirm saturation [20,39]. This sampling approach ensured comprehensive representation of perspectives across the organizational landscape. Participant demographic characteristics are summarized in Table 1.

**Table 1:** Participant demographics.

Demographic Category	n	%
**Total unique participants**	**111**	**100%**
**Data Source**		
Documentary comment contributors (one comment per employee)	87	78.4%
Semi-structured interview participants	24	21.6%
**Interview Participants (n = 24)**		
**Gender**		
Male	20	83.3%
Female	4	16.7%
**Organizational Level**		
Senior management	3	12.5%
Middle management	7	29.2%
Supervisory	6	25.0%
Professional staff	8	33.3%
**Functional Area**		
Operations	6	25.0%
Engineering	5	20.8%
IT/Cybersecurity	4	16.7%
Administration	5	20.8%
Finance	4	16.7%
**Organizational Tenure**		
< 5 years	7	29.2%
5-15 years	10	41.7%
> 15 years	7	29.2%
**Technical Expertise**		
IT professionals	5	20.8%
Moderate technical knowledge	11	45.8%
Limited technical knowledge	8	33.3%

### 3.3. Data collection procedures

Data collection proceeded through two complementary phases, both falling within an overall study window of 1 October 2025–31 January 2026. First, documentary data comprising 87 employee comments — one from each of 87 distinct employees — were systematically extracted from the organization’s internal electronic system. Comments were collected between 1 October 2025 and 17 December 2025. These comments were employees’ voluntary contributions to organizational dialogue on cybersecurity and information-safeguarding practices. Comments ranged from brief observations to extended reflections, collectively providing diverse perspectives on security challenges, experiences, and recommendations. The documentary data’s authenticity represents a methodological strength, capturing unfiltered employee voices in their natural organizational context.

Second, semi-structured interviews were conducted with 24 purposively selected employees over three months, between 1 November 2025 and 31 January 2026. The interview phase therefore overlapped with the documentary collection window, and both phases fall within the single post-incident feedback period described in Section 3.1; the two phases together span the four months from 1 October 2025–31 January 2026. An interview protocol guided conversations while permitting flexibility to explore emerging themes and follow participant narratives. Interview questions addressed participants’ understanding of information security threats, perceptions of organizational vulnerabilities, individual safeguarding behaviors, organizational culture regarding security, barriers to effective protection, training experiences, and recommendations for improvement. Interviews were conducted in participants’ preferred language (English or Arabic), audio-recorded with consent, and transcribed verbatim. Of the 24 interviews, nine were conducted in Arabic and subsequently translated into English by a professional bilingual translator specializing in organizational and technical terminology. To validate translation accuracy, a back-translation procedure was applied: a second independent bilingual researcher re-translated a randomly selected subset of three Arabic interview transcripts from the English version back into Arabic, and any discrepancies were reconciled by reference to the original Arabic transcripts, which were treated as authoritative. Minor terminological inconsistencies were resolved through consensus between the translator and the researcher.

### 3.4. Data analysis approach

Data analysis employed thematic analysis, a systematic approach for identifying, analyzing, and reporting patterns within qualitative data [39]. Thematic analysis was selected for its flexibility, accessibility, and capacity to generate rich insights from diverse data sources while remaining theoretically informed by Protection Motivation Theory. Analysis proceeded through iterative phases combining inductive and deductive elements, as summarized in Table 2. The specific coding techniques employed at each phase are detailed in Table 3.

**Table 2:** Thematic analysis process.

Phase	Description	Activities
1. Familiarization	Immersion in data to develop a deep understanding	Reading and re-reading all documentary comments and interview transcripts, noting initial impressions and patterns.
2. Initial Coding	Systematic identification of features relevant to research questions	Line-by-line coding of data; generating descriptive codes capturing semantic and latent meanings; organizing codes in NVivo software
3. Theme Development	Collating codes into potential themes	Grouping related codes, identifying patterns at broader levels of meaning, and considering relationships between codes and themes
4. Theme Refinement	Reviewing and refining theme coherence	Checking themes against coded data and the entire dataset, ensuring themes distinctly capture patterns, refining theme boundaries
5. Theme Definition	Clearly defining and naming themes.	Articulating the essence of each theme, identifying sub-themes, and ensuring alignment with research questions and the theoretical framework
6. Report Production	Producing scholarly analysis	Selecting compelling data extracts, integrating analysis with literature and theory, and crafting a coherent narrative addressing research objectives

**Table 3:** Coding techniques and steps.

Coding Level	Technique	Example
**First Cycle Coding**	Descriptive coding	*“backup practices,” “password behaviors,” “threat awareness”*
	In vivo coding	*“weakest link,” “everyone’s responsibility,” “cyber ethics”*
	Process coding	*“learning from incidents,” “developing habits,” “seeking training.”*
**Second Cycle Coding**	Pattern coding	*Grouping related first-cycle codes into broader categories*
	Focused coding	*Identifying the most significant codes requiring deeper analysis*
	Theoretical coding	*Connecting codes to PMT constructs (threat appraisal, coping appraisal)*
**Theme Development**	Thematic synthesis	*Integrating codes into coherent themes with sub-themes*
	Theory-driven organization	*Structuring themes around PMT dimensions and exploratory research questions*

Analysis began with open coding, in which both documentary comments and interview transcripts were coded line-by-line, generating initial codes that captured key concepts, experiences, and perspectives. This phase combined inductive coding (allowing themes to emerge from data) and deductive coding (informed by PMT constructs). Codes were systematically organized using NVivo qualitative analysis software, facilitating data management and pattern identification.

To establish coding reliability, a second independent researcher with expertise in qualitative organizational research coded a randomly selected 25% sub-sample of the dataset (approximately 22 documentary comments and six interview transcripts) using the same coding protocol. Inter-coder agreement was assessed using Cohen’s Kappa, yielding a coefficient of κ = 0.82, indicating strong agreement [40]. Disagreements (18% of initially coded segments) were resolved through discussion and consensus, with the coding framework refined accordingly. A summary codebook with code definitions and anchor examples for each code is provided as S1 File.

Subsequent analysis phases involved grouping related codes into broader categories, identifying connections between categories, and developing coherent themes capturing essential patterns across the dataset. Themes were continuously refined through iterative comparison with the raw data, ensuring they authentically represented participants’ perspectives while addressing the research questions. The analysis explicitly attended to PMT dimensions, examining how participant narratives reflected threat appraisals (perceived severity and vulnerability) and coping appraisals (response efficacy, self-efficacy, response costs). This theoretically informed analysis enabled a systematic exploration of how PMT constructs manifest in employees’ actual information-safeguarding experiences.

### 3.5. Ensuring research rigor

Multiple strategies enhanced research trustworthiness. Credibility was established through methodological triangulation combining documentary and interview data, enabling cross-validation of findings. Prolonged engagement with data through iterative analysis phases deepened interpretive insights. Transferability was enhanced through thick description of research context, participants, and findings, enabling readers to assess applicability to other settings. Dependability was strengthened through systematic documentation of methodological decisions, maintenance of detailed audit trails, and transparent reporting of analysis processes. Confirmability was pursued through reflexive awareness of researcher perspectives and potential biases, explicit grounding of interpretations in participant data, and presentation of contradictory evidence where present. As a researcher with professional and institutional familiarity with the Saudi Arabian organizational context, I maintained a dedicated reflexivity journal throughout the analysis process, systematically documenting interpretive assumptions, analytical decisions, and instances where my insider familiarity may have shaped data interpretation. This reflexive practice enhanced critical awareness of positionality and supported a more transparent, systematically examined analysis.

Researcher bias was managed through several concrete safeguards rather than reflexivity alone. First, an audit trail documented each analytical decision (e.g., code definitions, merges, and boundary cases) so that interpretive choices could be traced back to the raw data rather than to prior assumptions. Second, negative-case analysis was deliberately used: excerpts that appeared to contradict emerging themes were sought out and discussed in the reflexivity journal rather than set aside, and, where they could not be reconciled, they were reported as such in the Results. Third, during coding of segments touching on management or leadership behavior, where insider familiarity with the organization posed the greatest risk of interpretive bias, provisional codes were cross-checked against the second coder’s independent coding before being finalized, and any divergence was resolved through discussion rather than by the primary researcher’s judgment alone. Fourth, periodic memoing was used to separate the researcher’s prior professional impressions of the organization from participants’ own statements, flagging instances where an interpretation drew on outside knowledge so that this could be corrected against the transcript wording. These procedures were intended to keep insider familiarity operating as an analytic resource rather than as a source of unexamined bias.

### 3.6. Ethical considerations

The research adhered to rigorous ethical standards throughout all phases of data collection and analysis. Ethical approval was granted by the Research Ethics Committee at King Khalid University (Approval Reference: ECM#2026−1101) prior to commencing any data collection. Formal permission from the corporation’s senior leadership was obtained before accessing internal data or conducting employee interviews. Interview participants provided written informed consent after receiving comprehensive information about the research purposes, procedures, data confidentiality protections, their right to withdraw at any time without consequences, and their rights to voluntary participation. No minors participated in the study. Regarding the documentary data, the employee comments were obtained from the organization’s internal electronic system through formal institutional channels, with the required organizational permission granted. These data were fully de-identified prior to analysis: all names, specific job titles, department identifiers, and any other potentially identifying information were removed. The Research Ethics Committee at King Khalid University confirmed that access to de-identified, organizationally sanctioned internal communications under formal institutional permission (ECM#2026−1101) did not require individual consent waivers beyond the organizational-level approval obtained. Organizational identity remains anonymous throughout reporting, referenced generically as a large multinational energy corporation. All data were securely stored with access restricted solely to the researcher. These comprehensive ethical safeguards protected participant and organizational confidentiality while enabling rigorous scholarly inquiry into this strategically important topic.

## 4. Results

Thematic analysis of 87 employee comments — one contributed by each of 87 distinct employees — and 24 semi-structured interviews revealed five major themes that illuminate how employees within this multinational energy corporation perceive, experience, and respond to information-safeguarding imperatives. Each major theme encompasses several sub-themes, collectively providing comprehensive insights into the cognitive, behavioral, and contextual dimensions of employee information security practices. Results are organized by these major themes, with analysis demonstrating connections to constructs of Protection Motivation Theory and research propositions. Three denominators are used in this section and are stated explicitly wherever a proportion is reported: (i) the total number of coded references (approximately 490 coded segments across both data sources), used for the theme-level percentages given in the sub-section headings and in Table 4; (ii) the 87 documentary contributors, used where a finding derives from the documentary component alone; and (iii) the 24 interviewees, used where a finding derives from the interview component alone. Where a proportion refers to the study as a whole, the denominator is the full sample of 111 unique participants (87 documentary contributors and 24 interviewees). These denominators are distinct and are not interchangeable.

**Table 4:** Distribution of major themes.

Major Theme	% of Total Coded References	Participants Contributing (n of 111)	Documentary Contributors (n of 87)	Interviewees (n of 24)
Theme 1: Heightened Threat Awareness and Perceived Vulnerability	42%	97 (87%)	**76 (87%)**	**21 (88%)**
Theme 2: Individual Responsibility and Accountability Tensions	38%	93 (84%)	**72 (83%)**	**21 (88%)**
Theme 3: Organizational Culture and Management Commitment	33%	78 (70%)	**60 (69%)**	**18 (75%)**
Theme 4: Technical Systems Versus Human Factors	29%	71 (64%)	**55 (63%)**	**16 (67%)**
Theme 5: Knowledge Gaps, Training Needs, and Continuous Learning	26%	88 (79%)	**68 (78%)**	**20 (83%)**

Note. “% of Total Coded References” is calculated based on approximately 490 coded segments and can exceed 100% because a single segment may carry more than one code. “Participants Contributing” is calculated against the 111 unique participants, comprising 87 documentary contributors (the internal system permitted one submission per employee, so 87 comments correspond to 87 distinct employees) and 24 interviewees; the final two columns disaggregate that count by data source and sum to the combined figure. Coded-reference percentages and participant counts use different denominators and are not interchangeable.

### 4.1. Theme 1: Heightened threat awareness and perceived vulnerability (42% of coded references)

The most prominent theme emerging from the analysis concerned employees’ acute awareness of information security threats and their perceptions of organizational and personal vulnerability. This theme directly relates to PMT’s threat appraisal dimension, encompassing perceived severity of potential security breaches and perceived vulnerability to such threats.

*Sub-theme 1.1: Lessons Learned from Organizational Cyber Incidents*

A substantial majority of participants (72 of 111; 65%) explicitly cited the organization’s prior cyberattack as a watershed moment that transformed their security consciousness. Employees described how direct experience with a devastating cyber incident fundamentally altered their understanding of threat severity. As one employee commented:

*“Every time hackers are more aggressive, either just to play or sometimes with evil intentions behind their activities, we, everybody, must be suspicious about anything that may be a potential exposure to lose information integrity.”*

Another reflected,

*“New people are coming every day, and many did not experience the cyber-attack last year. So for all the newbies... Back up your work on the network.”*

Interview participants elaborated on how organizational crisis crystallized abstract threats into tangible realities. A senior operations manager stated:

*“Before the attack, cybersecurity was something IT worried about. After experiencing data loss and operational disruption firsthand, everyone understood that we are all vulnerable. It was not theoretical anymore—we lived it.”*

This experiential learning dramatically elevated perceived threat severity, with employees recognizing that potential consequences extended beyond technical inconvenience to include operational disruption, data loss, reputational damage, and national security implications.

*Sub-theme 1.2: Recognition of Evolving Threat Landscape*

Employees demonstrated a sophisticated understanding of the dynamic, escalating nature of cyber threats. Fifty of the 87 documentary comments (57%) referenced increasing threat sophistication, persistence, and diversification. One employee observed,

*“In today’s high-tech and fast-paced modern world, everything is possible, and the intruders and culprits are taking this opportunity. They are just roaming around in cyberspace. Viruses, spam, phishing, hackers, and spyware are just waiting for our vulnerability.”*

Participants recognized threats emanating from multiple sources, including state-sponsored actors, criminal organizations, and opportunistic hackers. An IT professional noted during interviews,

*“We are not just facing random hackers anymore. These are sophisticated, well-resourced adversaries specifically targeting energy infrastructure. They study our systems, probe for weaknesses, and wait for the right moment.”*

This recognition of persistent, targeted threats heightened perceived vulnerability, particularly among employees handling sensitive information or managing critical systems.

*Sub-theme 1.3: Vulnerability Across Multiple Information Channels*

Notably, employees recognized vulnerability extending beyond digital systems to encompass physical documents, verbal communications, and social engineering tactics. Forty-one of the 87 documentary comments (47%) addressed physical information security, reflecting a comprehensive understanding of threats. As one employee stated,

*“Information thieves are not just on the web, but are also involved in daily activities, looking for information in hard copy. Printed records are usually overlooked in this protection.”*

Participants identified vulnerabilities, including unattended printed documents, unsecured file cabinets, confidential conversations in public spaces, and improper disposal of sensitive materials. An administrative manager recounted,

*“I notice that many employees left the printed paper without taking it. If they do not need them, they can shred them rather than keep them there, so anyone passing by can read them.”*

This holistic vulnerability awareness demonstrates sophisticated threat appraisal encompassing diverse information exposure vectors, though subsequent themes reveal inconsistencies between awareness and consistent protective behaviors.

*Sub-theme 1.4: Organizational Consequences and National Security Implications*

Employees articulated an understanding that information security breaches carry consequences that transcend individual or departmental impacts, affecting organizational reputation, competitive position, and national security interests. One employee stated,

*“Upstream information items are a national asset. The risk of exposure varies from one information asset to another. Safeguarding this information is not a trivial task and requires an ongoing and collaborative effort.”*

Interview participants working in strategic roles expressed particular awareness of national security dimensions. A senior engineering manager explained,

*“Our exploration data, production technologies- these are not just company assets—they are national strategic resources. A breach does not just hurt the company; it potentially compromises national interests.”*

This recognition of multilevel consequences amplifies perceived threat severity, though subsequent analysis reveals that elevated awareness does not automatically translate into consistent protective behaviors across all employee segments.

### 4.2. Theme 2: Individual responsibility and accountability tensions (38% of coded references)

The second major theme concerned employees’ understanding of individual responsibilities for information protection and tensions surrounding accountability. This theme relates to both PMT’s coping appraisal (particularly self-efficacy regarding protective actions) and the dimensions of organizational culture.

*Sub-theme 2.1: “Everyone’s Responsibility” Rhetoric*

A dominant narrative, appearing in 62 of the 87 documentary comments (71%), emphasized that information safeguarding is everyone’s responsibility, not solely the IT department’s. As one employee succinctly stated,

*“Safeguarding information is everyone’s responsibility. Everyone has a responsibility for safeguarding information.”*

Another elaborated,

*“What I believe is that everyone is responsible for his/here conduct both in and out of the workplace. People know that opening any email attachment or surfing any site that has the potential to cause harm to our network is a bad idea.”*

This rhetoric reflects organizational messaging promoting distributed security responsibility. However, interview data revealed complexity beneath this surface consensus. While employees intellectually accepted shared responsibility, many struggled to translate this principle into consistent daily behaviors. A mid-level manager admitted,

*“We all say it is everyone’s job, but in practice, people still think ‘IT handles security.’ There is a gap between what we say and what we actually do.”*

*Sub-theme 2.2: Role Ambiguity and Boundary Questions*

Despite widespread endorsement of shared responsibility, 11 of the 24 interviewees (46%) expressed uncertainty about which responsibilities fall to individuals, the organization, or the IT department. Employees questioned where individual accountability ends and organizational systems should compensate for human limitations. One professional staff member asked,

*“How much can we reasonably expect individuals to remember—complex passwords, backup schedules, security procedures? At what point should systems automatically protect us?”*

This ambiguity manifested in tensions around technology usage. Employees recognized that behaviors such as using personal devices, accessing external websites, or storing data on local drives could compromise security. Nevertheless, these practices persisted when organizational systems created workflow obstacles. An operations supervisor explained,

*“Official policy says one thing, but to actually get work done efficiently, sometimes you need workarounds. Where is the line between being pragmatic and being irresponsible?”*

Not all employee perspectives endorsed the prevailing security approach. A notable minority of participants expressed skepticism or resistance toward specific security measures, providing important analytical balance. One employee commented, “There are too many rules, and they keep changing. I sometimes wonder whether all these procedures actually make us safer or create the appearance of security.” Another stated: “When security restrictions prevent me from completing my work efficiently, I question whether those designing these policies truly understand our operational realities.” These dissenting perspectives, while less prevalent than supportive accounts, are analytically significant for capturing the full spectrum of employee responses to security imperatives and are consistent with Beautement et al.’s [6] concept of the compliance budget, in which employees weigh the perceived costs of security compliance against operational demands and benefits.

*Sub-theme 2.3: Self-Efficacy Variations Across Employee Segments*

Analysis revealed substantial variation in employees’ confidence regarding their capability to execute effective information protection behaviors—a core PMT self-efficacy dimension. IT professionals and other interviewees reporting high technical proficiency (8 of 24; 33%) expressed high self-efficacy, demonstrating detailed knowledge of security practices and confidence in implementing them. As an IT professional stated,

*“I personally believe in ‘IT Security’ and have been proactive about it in my personal life before I embarked on ‘security’ as a new career. I delivered awareness sessions for over 2000 people as of now.”*

Conversely, the 8 interviewees with limited technical knowledge (33%; see Table 1) reported lower self-efficacy and greater anxiety regarding security responsibilities. An administrative employee admitted,

*“I know I should back up regularly, create strong passwords, and be careful with emails. Nevertheless, honestly, I am not confident I am doing it correctly. The technical language confuses me, and I worry I am missing something important.”*

This self-efficacy gap represents a critical vulnerability, as employees lacking confidence may avoid protective behaviors or implement them incorrectly, despite recognizing the severity of the threat.

*Sub-theme 2.4: Accountability and Consequences*

Participants expressed mixed perspectives on whether accountability mechanisms effectively motivate protective behaviors. Thirty-three of the 87 documentary comments (38%) referenced the need for accountability, with some advocating stronger enforcement while others emphasized positive reinforcement. One employee suggested,

*“We have to promote a well-organized campaign to have every user accountable for his/her actions. This company is packed with reliable individuals who are willing to safeguard themselves.”*

However, interview participants revealed that accountability systems often remained abstract or were applied inconsistently. A middle manager observed,

*“There are policies about security violations, but enforcement seems random. Some violations get addressed seriously, while others are ignored. This inconsistency sends mixed messages about whether leadership actually prioritizes security.”*

The perceived gap between stated accountability expectations and actual consequences moderates employees’ motivation to consistently engage in protective behaviors, reflecting PMT’s response cost dimension, in which perceived effort may exceed perceived benefit when accountability remains uncertain.

### 4.3. Theme 3: Organizational culture and management commitment (33% of coded references)

The third major theme concerned organizational security culture and the critical role of management commitment in shaping employee behaviors. This theme addresses contextual factors that moderate the relationships among threat awareness, coping appraisals, and actual protective behaviors.

*Sub-theme 3.1: Post-Incident Cultural Transformation*

Employees described significant cultural shifts following the organization’s major cyber incident. Sixty of the 111 participants (54%) noted elevated organizational attention to security, as evidenced by increased communication, training initiatives, policy updates, and leadership messaging. One employee observed,

*“Thank you for presenting this topic. Information security is an area of major concern in our company; without it, an institution’s private data and intellectual property could be compromised.”*

However, participants questioned whether heightened attention would sustain over time or prove temporary. A veteran employee cautioned,

*“Right after the attack, security was top priority. Everyone talked about it, and management emphasized it constantly. Nevertheless, I have seen this before—attention fades, people get complacent, and we slip back into old habits until the next crisis.”*

This concern reflects awareness that genuine cultural transformation requires sustained commitment rather than reactive episodes of heightened awareness.

*Sub-theme 3.2: Management Commitment Signals*

Employees emphasized that visible management commitment powerfully influences security culture. Fifty-four of the 87 documentary comments (62%) referenced management’s role in establishing security priorities, modeling behaviors, and allocating resources. As one employee stated,

*“Management championship shall help to drive end users’ adoption. Furthermore, information governance needs to be formalized at the corporate level, which covers the content classification, retention, and information compliance.”*

Interview participants identified specific management behaviors that signal authentic commitment: personally adhering to security protocols, acknowledging security concerns in decision-making, allocating budgets for security initiatives, recognizing employees who demonstrate exemplary security practices, and publicly addressing security violations regardless of the perpetrator’s seniority. Conversely, employees noted that when management rhetoric contradicted behavior, such as leaders bypassing security procedures for convenience, it immediately undermined credibility. A supervisor stated,

*“When senior management follows the same rules they expect from us, it sends a powerful message. Nevertheless, when they circumvent policies because they are ‘too busy,’ employees notice and figure policies are not really that important.”*

*Sub-theme 3.3: Peer Influence and Social Norms*

Participants described how colleague behaviors and social norms within work units substantially influenced individual security practices. Twelve of the 24 interviewees (50%) addressed peer influence, noting that they modeled behaviors observed among colleagues and felt social pressure to conform to group norms. An engineering professional explained,

*“In my unit, everyone takes security seriously. We remind each other to lock computers, we do not leave documents unattended, and we call out risky behaviors. That culture keeps everyone sharp.”*

Conversely, employees working in units with lax security norms described pressure to conform to non-compliant behaviors to avoid appearing paranoid or uncooperative. A professional staff member recounted,

*“My previous department was casual about security. If you insisted on following every protocol, colleagues viewed you as uptight or distrustful. There was social pressure to be ‘reasonable’ and not overthink security, which really meant accepting unnecessary risks.”*

These findings underscore that security culture operates through micro-level social dynamics within work units, not merely through organization-wide policies, and that peer norms can either reinforce or undermine organizational security objectives.

*Sub-theme 3.4: Integration of Security into Daily Operations*

Employees emphasized that an effective security culture requires integrating protection into everyday workflows rather than treating security as separate, additional tasks. Forty-six of the 111 participants (41%) noted tensions between security requirements and operational efficiency. One employee observed,

*“One year down the line, I feel we have to draw a moderate line between usability and security. Too many internet restrictions negatively impact smooth operations and access to the information that you need.”*

Interview participants described how poorly designed security measures that excessively burden workflows prompt workarounds, inadvertently creating new vulnerabilities. A manager explained,

*“If security procedures are so cumbersome that they triple the time required for routine tasks, people will find shortcuts. We need security integrated smoothly into workflows, making the secure way also the easy way.”*

This perspective aligns with research on security usability trade-offs and highlights that organizational culture must balance protection imperatives with operational realities to achieve sustainable security behaviors.

### 4.4. Theme 4: Technical systems versus human factors (29% of coded references)

The fourth major theme addressed the interplay between technical security controls and human behavioral factors, revealing employee perspectives on optimal security approaches.

*Sub-theme 4.1: Technical Controls as Foundation*

Employees recognized the technical security infrastructure as essential foundational protection. Forty-five of the 87 documentary comments (52%) referenced technical measures, including firewalls, antivirus software, intrusion detection systems, access controls, and encryption. As one employee stated,

*“Effective IT computing resources, security, and network traffic monitoring are the best ways to ensure that information in transit or stored does not get leaked.”*

Participants generally expressed confidence in organizational technical capabilities while acknowledging that technology alone cannot guarantee security. An IT professional noted,

*“We have strong technical defenses—enterprise-grade firewalls, sophisticated monitoring systems, layered access controls. Nevertheless, technology is only effective if users do not circumvent it through risky behaviors.”*

This perspective reflects a balanced understanding that technical and human elements must work in concert to achieve comprehensive protection.

*Sub-theme 4.2: Humans as the “Weakest Link”*

A prevalent narrative, present in 58 of the 87 documentary comments (67%), identified human behavior as the most significant security vulnerability despite the presence of robust technical controls. One employee bluntly stated,

*“Regardless of how much IT puts into protection systems and measures, WE, the human users, remain the weakest link in the chain! Unfortunately, phishing attacks and social engineering remain successful and effective hacking mechanisms.”*

Interview participants elaborated on human vulnerabilities, including susceptibility to social engineering, password weaknesses, careless handling of sensitive information, and failure to follow security protocols. A cybersecurity professional explained,

*“The most sophisticated attack we analyzed did not exploit technical vulnerabilities—it exploited human psychology. An employee clicked a convincing phishing link, providing access that bypassed all our technical defenses. Technology cannot fix human judgment errors.”*

This widespread recognition of human factors as critical vulnerabilities underscores the importance of addressing behavioral dimensions alongside technical measures.

*Sub-theme 4.3: Automation and User-Friendly Security*

Employees advocated for increased automation and user-friendly security design to reduce reliance on consistent human vigilance. Forty-nine of the 111 participants (44%) suggested automated backup systems, simplified authentication methods, and intuitive security interfaces. One employee proposed:

*“There should be a common folder on a pc where we should save respective essential data, and that data needs to be backed up automatically in the department backup server, like an image, to avoid unexpected damage regularly.”*

Another employee who attempted to implement automated backups encountered obstacles:

*“I tried to use the Windows backup functionality to automate backing up and make it less prone to forgetfulness. I found out I needed admin rights, but the functionality is not enabled! It would be great if IT activates this functionality or an application that enables the user to automate when and what to back up regularly.”*

These perspectives reflect PMT’s response cost dimension—employees seek protective mechanisms requiring minimal ongoing effort and cognitive burden. By automating security functions and designing intuitive interfaces, organizations can reduce response costs while enhancing the effectiveness of protection. However, participants acknowledged that complete automation remains impossible, necessitating sustained attention to human behavioral factors.

*Sub-theme 4.4: Balance Between Control and Trust*

Participants expressed tensions between security controls and employee autonomy, questioning the optimal balance between restrictions and trust. Nine of the 24 interviewees (38%) addressed this tension. Employees described frustration with overly restrictive controls that blocked legitimate work activities, while acknowledging the need for reasonable restrictions. One employee lamented:

*“Sadly, with the new procedures in protecting our ‘privacy’ & ‘information,’ a lot of good websites- that are work-related- are banned, and personal Emails are blocked. So yes, I understand all this, and it is well justified, but at the same time, maybe we should be offered a free hotspot to connect to the company’s network!”*

Interview participants suggested that excessive restrictions signal organizational distrust, potentially diminishing employee morale and commitment to security objectives. A mid-level manager proposed:

*“Security should not be about assuming employees are threats. It should be about empowering people to work safely. When every control feels like surveillance rather than support, it breeds resentment rather than cooperation.”*

These perspectives highlight the need for organizational security approaches that balance protection with respect for employee professionalism and judgment, cultivating collaborative rather than adversarial security cultures.

### 4.5. Theme 5: Knowledge gaps, training needs, and continuous learning (26% of coded references)

The fifth major theme concerned employees’ recognition of knowledge gaps and their expressed need for enhanced training and ongoing education on information safeguarding practices. This theme directly relates to PMT’s coping appraisal, particularly self-efficacy and response efficacy dimensions.

*Sub-theme 5.1: Awareness of Personal Knowledge Gaps*

Employees demonstrated reflexive awareness of their own knowledge limitations regarding information security. Sixty-four of the 111 participants (58%) acknowledged insufficient understanding of specific security practices, appropriate responses to threats, or rationales for security requirements. One employee candidly stated:

*“As a person who is not an expert in cyber, I think the first lesson is always to back up your data. In this way, I will be sure that my data will never be lost.”*

Interview participants with limited technical backgrounds expressed particular anxiety about knowledge gaps. An administrative professional admitted,

*“I hear terms like ‘encryption,’ ‘two-factor authentication,’ ‘VPN,’ but honestly do not fully understand what they mean or how to use them properly. I follow instructions but do not really comprehend the underlying logic, which makes me doubt whether I am protecting information adequately.”*

This awareness motivates learning while highlighting that current knowledge remains insufficient for employees to consistently and confidently execute effective protective behaviors.

*Sub-theme 5.2: Importance of Education and Awareness Programs*

A substantial majority of the documentary comments (64 of 87; 74%) emphasized the critical importance of ongoing education and awareness initiatives. Employees repeatedly advocated for continuous training rather than one-time sessions. As one employee stated:

*“Ongoing educational sessions and programs on information safeguarding are required to raise awareness among all employees, especially newcomers to the company. This is the responsibility of all organizations and employees as well.”*

Participants recognized that effective education must address diverse learning needs across employee populations. One employee proposed:

*“I think that through proper education and awareness, the company will keep a work environment protected, safe, and secure.”*

Another emphasized reaching beyond technical staff:

*“If continuous education and awareness of the company’s employees take place from time to time, the company information will be kept safe and secure.”*

Interview participants described a desire for practical, role-specific training that addresses actual situations encountered in their work, rather than generic presentations. An operations supervisor suggested:

*“Training should show us realistic scenarios we might actually face—suspicious emails that look legitimate, decisions about sharing information with contractors, and handling sensitive documents in shared workspaces. We need practical guidance for real situations, not just theory.”*

*Sub-theme 5.3: Lessons Learned and Best Practice Sharing*

Employees expressed frustration that lessons from organizational security incidents were insufficiently communicated across the organization, resulting in missed learning opportunities. Thirty-seven of the 87 documentary comments (43%) addressed this concern. One employee observed,

*“Yet lessons learned as a result of the last cyber-attack are not communicated openly to the rest of the company. The big picture of protecting company information is not clear to all employees, as outsiders are part of our daily communication and business.”*

Participants advocated for systematic sharing of incident analyses, emerging threats, and effective protective practices across the organization. An engineering professional suggested:

*“When incidents happen, instead of hushing them up, we should use them as teaching moments. What went wrong? How was it detected? What should others watch for? This organizational learning is invaluable.”*

This perspective aligns with organizational learning theory, which emphasizes that collective intelligence about threats and responses enhances the overall security posture.

*Sub-theme 5.4: Peer-to-Peer Learning and Grassroots Awareness*

Beyond formal training programs, employees recognized the value of peer-to-peer learning and grassroots awareness initiatives. Seven of the 24 interviewees (29%) described informal knowledge sharing among colleagues as a valuable learning mechanism. One security-conscious employee championed peer education:

*“Therefore, I suggest that anyone who wants to contribute conduct an awareness session within their organization! It is super easy!! Attend the training/awareness sessions conducted regularly by IT, or request one from your respective IT Division in your area. It only takes a 30-minute presentation to save lives potentially!”*

Interview participants described how colleagues with security expertise could effectively translate technical concepts into accessible language and address specific departmental challenges. A professional staff member explained:

*“When our unit’s technically savvy colleague explains security in terms of our actual work processes, it makes much more sense than generic IT presentations. He speaks our language and addresses our specific situations.”*

These findings suggest that cultivating distributed security expertise and encouraging peer education can complement formal training programs, enhancing knowledge diffusion throughout the organization.

*Sub-theme 5.5: Desire for Practical Guidance*

Employees expressed a desire for concrete, actionable guidance on implementing security practices rather than abstract principles. Fifty-four of the 111 participants (49%) requested practical instructions, checklists, and tools facilitating protective behaviors. One newcomer pleaded:

*“Having simple instructions on backing up data would be helpful for us newbies.”*

Documentary comments revealed employees seeking specific guidance on diverse topics: appropriate backup procedures, secure document handling, confidentiality protocols for various communication channels, and responses to suspicious activities. One employee provided detailed suggestions covering telephone procedures, fax machine protocols, copy machine behavior, email practices, computer security, and paper document handling, demonstrating an appetite for comprehensive, practical guidance.

Interview participants emphasized that practical guidance should be easily accessible at the point of need rather than buried in lengthy policy documents. A manager proposed:

*“We need quick reference guides, visual reminders at workstations, and simple flowcharts for common decisions. When you are in the moment deciding whether to share information or how to dispose of a document, you need instant guidance, not a 50-page policy manual.”*

These perspectives highlight that enhancing employees’ response efficacy and self-efficacy requires providing accessible, practical tools that support protective behaviors in real-world work contexts.

### 4.6. Quantitative summary of thematic analysis

Analysis of the coded data reveals the following distribution of themes and sub-themes across the dataset. In Table 4, the column “% of Total Coded References” represents the proportion of all coded data segments (approximately 490 coded segments across both data sources) attributed to each theme; these percentages sum to more than 100% because a single data segment may be coded under more than one theme. The column “Participants Contributing” indicates the number of individual participants who contributed at least one data segment coded under that theme, expressed as a percentage of the full sample of 111 unique participants. That denominator comprises 87 documentary contributors — the internal system permitted one submission per employee, so the 87 comments were contributed by 87 distinct employees (Section 3.2) — together with the 24 interviewees. Table 4 additionally disaggregates each theme by data source, so that the documentary and the interview contribution to each theme can be inspected separately and the two denominators (87 and 24) are visible alongside the combined denominator (111). For Theme 1, for example, n = 97 of 111 participants (87.4%), reported as 87%; that figure comprises 76 of the 87 documentary contributors (87%) and 21 of the 24 interviewees (88%). Coded-reference percentages and participant counts are therefore reported on separate, explicitly stated denominators and are not interchangeable.

### 4.7. Conceptual framework: Integrating findings with protection motivation theory

Based on thematic analysis, a conceptual framework emerges that illustrates how PMT constructs manifest in employees’ information-safeguarding experiences and how contextual factors moderate these relationships (Fig 1).

**Fig 1:**
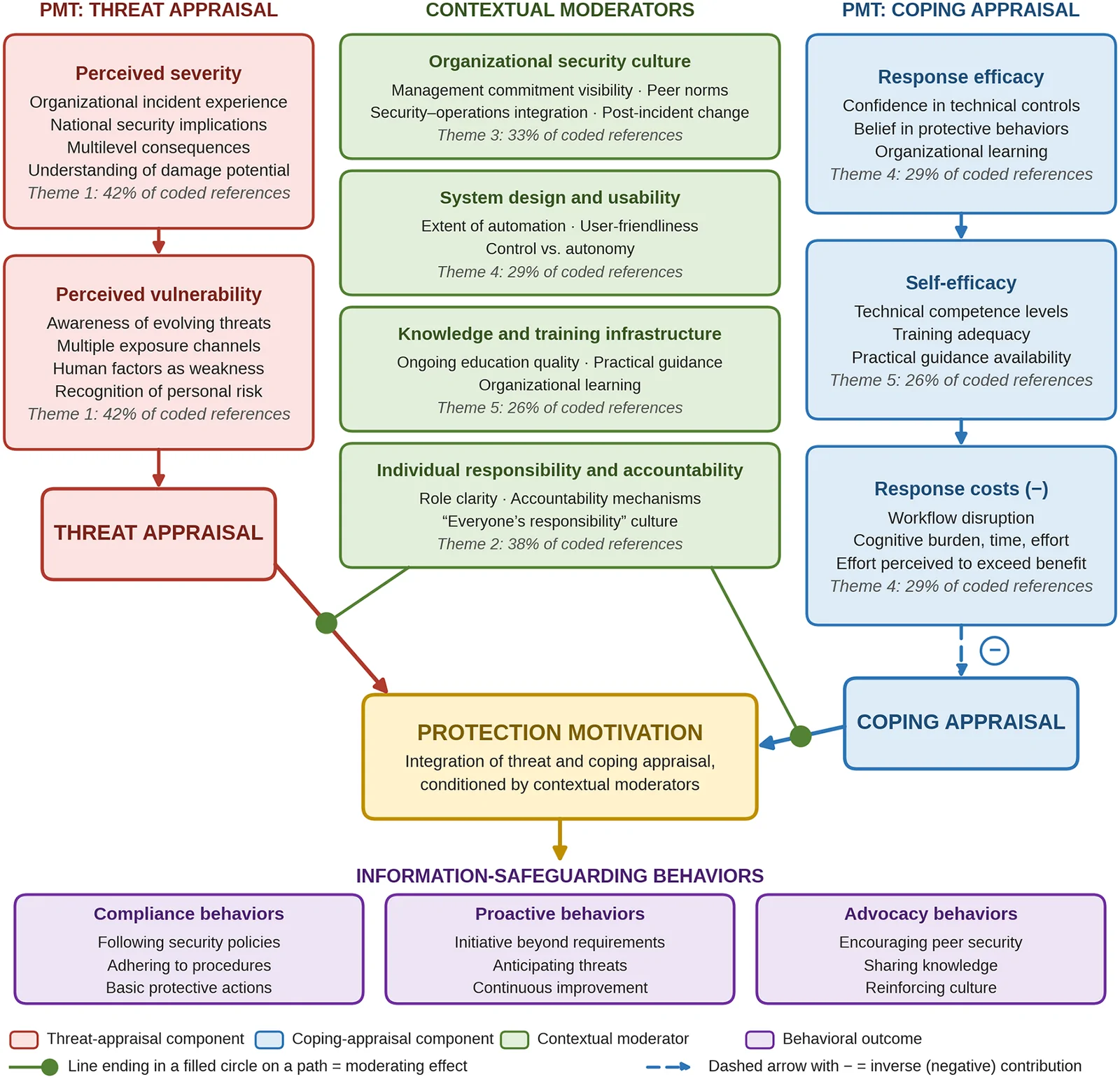
Conceptual framework of employee information-safeguarding behavior grounded in Protection Motivation Theory. Threat-appraisal components (red) and coping-appraisal components (blue) are integrated in protection motivation (amber), which is expressed in compliance, proactive, and advocacy safeguarding behaviors (purple). Contextual moderators (green) condition the appraisal-to-motivation paths, shown by arrows terminating in filled circles on those paths; the dashed arrow with a minus sign denotes the inverse contribution of response costs to coping appraisal.

The framework integrates the Protection Motivation Theory (PMT) threat and coping appraisal components with contextual moderators to explain employees’ information-safeguarding behaviors. Threat appraisal comprises perceived severity and perceived vulnerability, while coping appraisal comprises response efficacy, self-efficacy, and response costs. The four contextual factors — organizational security culture, system design and usability, knowledge and training infrastructure, and individual responsibility and accountability — are specified as moderators rather than mediators, because the data indicate that they condition the strength of the relationship between employees’ appraisals and their protective behavior rather than transmitting that relationship as intervening variables. This is represented using the standard moderation convention: the arrows from the contextual block terminate on the appraisal-to-protection-motivation paths, each termination marked by a filled circle, rather than entering the protection motivation node as parallel inputs. Response costs contribute inversely to coping appraisal, consistent with PMT; this directional distinction is represented visually by a dashed arrow carrying a minus sign, and is recorded in the legend. A single fill color is applied to every box within a category, so that color alone distinguishes the four categories: PMT threat appraisal components (red), PMT coping appraisal components (blue), contextual moderators (green), and behavioral outcomes (purple). Protection motivation, shown in amber as the integrating node, is expressed in compliance, proactive, and advocacy information-safeguarding behaviors.

Fig 1 illustrates that high threat awareness alone proves insufficient for consistent protective behaviors without corresponding high coping appraisals and supportive contextual conditions. Conversely, even employees with high self-efficacy may exhibit inconsistent behavior when organizational culture sends ambiguous signals or when system design imposes excessive response costs. This integrated model explains variation in employee security behaviors observed across the dataset and identifies multiple intervention points to enhance organizational security.

## 5. Discussion

The findings illuminate the complex interplay among cognitive, behavioral, organizational, and contextual factors that shape employee information-safeguarding practices within this multinational energy corporation. This discussion interprets the findings in relation to the existing literature and Protection Motivation Theory, addresses the research propositions, and examines the implications for theory and practice.

### 5.1. Threat awareness and the paradox of incomplete translation to behavior

Findings are consistent with and extend Exploratory Research Question 1, demonstrating that employees who perceived greater severity and vulnerability to information security threats exhibited greater awareness of safeguarding imperatives. The organization’s prior cyber incident served as a critical catalyst, elevating threat appraisals across the workforce, consistent with PMT’s prediction that experiencing threatening events intensifies perceived severity and vulnerability. However, findings reveal a crucial paradox: heightened threat awareness does not automatically or uniformly translate into consistent protective behaviors.

This paradox extends existing literature examining the “knowing-doing gap” in information security contexts. While Bulgurcu et al. [10] found security awareness predicted compliance intentions, the present research reveals that awareness represents a necessary but insufficient condition for actual behavioral change. Employees articulated a sophisticated understanding of diverse threats, including technical vulnerabilities, social engineering tactics, and physical exposure of information—yet simultaneously acknowledged inconsistent execution of protective practices. This disconnect reflects what Beautement et al. [6] characterized as pragmatic trade-offs between security ideals and operational realities, in which competing demands, cognitive limitations, and habitual behaviors constrain the behavioral translation of threat awareness.

The findings advance PMT by demonstrating that the influence of threat appraisal on behavior depends critically on contextual factors that moderate this relationship. Specifically, organizational culture, peer norms, management commitment signals, and system usability emerged as powerful moderators determining whether threat awareness translates into action. This suggests that PMT applications in organizational settings must attend to contextual conditions that either facilitate or impede the threat-awareness-to-behavior pathway, thereby extending theory beyond its original individual-level health-behavior focus.

### 5.2. Self-efficacy variability and the critical role of coping appraisal

Findings are consistent with and extend Exploratory Research Question 2, showing that employees’ information protection behaviors were significantly influenced by self-efficacy beliefs and perceived response efficacy, aligning with PMT’s coping appraisal dimension. However, the research reveals substantial heterogeneity in self-efficacy among employee segments, leading to differing security capabilities and vulnerabilities.

IT professionals and technically sophisticated employees demonstrated high self-efficacy, expressing confidence in their ability to understand threats and implement effective protective measures. Conversely, employees with limited technical backgrounds exhibited lower self-efficacy and anxiety regarding security responsibilities, despite recognizing the importance of threats. This self-efficacy gradient creates organizational vulnerability, as security effectiveness may be disproportionately affected by the lowest-capability employees rather than by average organizational capabilities. The finding aligns with Johnston and Warkentin’s [19] demonstration that self-efficacy significantly predicts security compliance intentions, while extending understanding by revealing how self-efficacy disparities across employee populations create systemic vulnerabilities.

Response efficacy beliefs also varied substantially. Employees generally expressed confidence in the organization’s technical controls but questioned whether individual behaviors meaningfully enhanced security beyond these safeguards. This skepticism about the efficacy of personal responses may undermine motivation to maintain consistent protective behaviors. The finding resonates with Karlsson et al.’s [11] observation that employees view security primarily as an organizational rather than an individual responsibility, suggesting that cultivating strong response efficacy beliefs requires explicitly demonstrating how individual behaviors complement technical controls to create comprehensive protection.

The research reveals that response costs—the third coping appraisal dimension—substantially constrain protective behaviors even when threat awareness and efficacy beliefs are firm. Employees described security requirements that imposed workflow disruptions, cognitive burdens, and time penalties, prompting workarounds and non-compliance. This finding corroborates Sasse et al.‘s [5] research on security-usability trade-offs, demonstrating that protective behaviors compete with operational efficiency pressures. Organizations must address response costs by integrating user-friendly security design and workflows to achieve sustainable compliance.

### 5.3. Organizational culture as critical moderator

Findings are consistent with and extend Exploratory Research Question 3, demonstrating that organizational culture and management commitment substantially moderate the relationships between security awareness and actual protective behaviors. Employees emphasized that visible management commitment, peer norms, and the integration of security into operations strongly influenced whether security knowledge translated into consistent action.

Management commitment emerged as particularly influential. Employees closely observed whether leaders personally adhered to security protocols, allocated resources to security initiatives, acknowledged security considerations in decision-making, and consistently addressed violations, regardless of the perpetrator’s status. Discrepancies between management rhetoric and behavior immediately undermined credibility and employee motivation. This finding aligns with research on organizational culture, which emphasizes that values become embedded through leaders’ behaviors rather than pronouncements [41]. Specifically in security contexts, Da Veiga and Eloff [26] demonstrated that organizations with strong security cultures experience fewer incidents, supporting the present finding that culture is a critical implementation mechanism for security objectives.

The research reveals tensions between security requirements and operational efficiency that organizational culture must navigate. When security measures excessively burden workflows, employees develop workarounds that create new vulnerabilities. Sustainable security culture requires integrating protection into daily operations, making secure behaviors the path of least resistance rather than an additional imposition. This finding reinforces the importance of security usability design while highlighting that culture determines whether employees view security as integral to operational excellence or as an obstruction to be circumvented.

### 5.4. Contextual factors and the saudi energy sector

Findings are consistent with and extend Exploratory Research Question 4, revealing that contextual factors specific to the Saudi Arabian energy sector distinctly shaped employees’ perspectives on information safeguarding. The strategic national importance of energy infrastructure created heightened security consciousness extending beyond typical corporate concerns. Employees explicitly recognized that information assets were national resources whose protection transcended organizational interests and encompassed national security imperatives.

The research also reveals cultural factors creating implementation challenges. The collectivist orientation of Saudi Arabian organizational culture — as characterized in cross-cultural management scholarship [42,43] — may emphasize group harmony and hierarchical deference, potentially constraining individuals’ willingness to challenge colleagues’ risky behavior or to report violations. This interpretation is offered tentatively; individual variation was evident within the sample, and cultural generalizations carry inherent risks of oversimplification. While the “everyone’s responsibility” rhetoric was widely endorsed, implementing accountability faced obstacles when violations involved senior personnel or when enforcement might disrupt group cohesion. This finding highlights how cultural values can simultaneously enhance (through collective security consciousness) and constrain (through reluctance to enforce individual accountability) the effectiveness of information protection.

The organization’s prior cyber incident experience provided a unique contextual influence, creating a workforce with a visceral understanding of threat severity and organizational vulnerability—this experiential knowledge distinguished study participants from typical employee populations whose threat awareness remains more abstract. While elevated awareness following incidents represents an opportunity for cultural transformation, findings also revealed concerns about awareness decay over time as incident memory fades. Sustaining elevated security consciousness requires ongoing reinforcement rather than relying solely on memory of incidents.

### 5.5. Human factors as persistent vulnerability despite technical sophistication

A striking finding was employees’ recognition that human behavior constitutes the most significant security vulnerability despite robust technical controls. This finding corroborates extensive research identifying humans as the “weakest link” in organizational security architectures [5,6]. However, the present research advances understanding by documenting this recognition from employees themselves rather than security professionals, revealing widespread reflexive awareness of human vulnerabilities.

The finding that employees strongly advocated for automated security mechanisms and user-friendly design reflects a desire to reduce reliance on consistent human vigilance. From a PMT perspective, automation reduces response costs while potentially enhancing response efficacy by ensuring protective actions occur reliably regardless of individual memory or motivation. Organizations should prioritize security architectures that account for human fallibility and compensate with automated safeguards, reserving human judgment for decisions that require contextual interpretation rather than routine protective actions.

### 5.6. Knowledge gaps and the continuous learning imperative

Findings revealed substantial knowledge gaps in specific security practices despite elevated general awareness, underscoring continuous learning as an imperative rather than a one-time achievement. Employees demonstrated reflexive awareness of their knowledge limitations and expressed a desire for practical guidance, ongoing training, and accessible resources that support protective behaviors at the point of need.

This finding aligns with research on adult learning, which emphasizes that effective training must be practical, relevant, and accessible in real-world work contexts [44]. Generic security presentations that review abstract threats and policies prove less effective than role-specific guidance that addresses the actual situations employees encounter. Interview participants specifically requested scenario-based training that illustrates realistic decisions about information sharing, suspicious activity recognition, and appropriate protective responses in their work contexts.

Notably, employees expressed frustration that lessons from organizational incidents were insufficiently communicated across the organization, resulting in missed learning opportunities. Systematic incident-analysis sharing, coupled with discussion of emerging threats and effective practices, could enhance collective organizational intelligence regarding security. This approach aligns with organizational learning theory, which emphasizes that knowledge creation and diffusion processes determine organizational adaptive capacity [45]. Within security contexts, cultivating learning cultures that openly discuss vulnerabilities, share near-miss experiences, and collectively analyze threats can enhance organizational resilience.

## 6. Research contributions, limitations, and future research

### 6.1. Research contribution and implications

#### 6.1.1. Theoretical contributions.

This research makes several theoretical contributions to information security scholarship by extending PMT to an underexplored sector and cultural context, while acknowledging that the exploratory single-case design limits the scope for direct generalization beyond this setting. First, it extends the application of Protection Motivation Theory to organizational information security contexts within the critical energy infrastructure sector, demonstrating PMT’s explanatory power while revealing necessary theoretical extensions. Specifically, the research illuminates how contextual factors, including organizational culture, management commitment, peer norms, and system usability, substantially moderate the relationships between PMT’s core constructs (threat appraisal and coping appraisal) and actual behaviors. This extension advances PMT from its origins in individual-level health behavior toward a comprehensive sociotechnical theory that accommodates organizational complexities.

Second, the research develops an integrated conceptual framework that demonstrates the complex interactions among threat awareness, self-efficacy, response efficacy, response costs, and contextual moderators that shape information-safeguarding behaviors. This framework provides a theoretical architecture for understanding why threat awareness alone is insufficient for behavioral change and identifies multiple intervention points to enhance security effectiveness. The framework serves as a foundation for future theory development and empirical testing across diverse organizational contexts.

Third, the research contributes to organizational culture theory by demonstrating specific mechanisms by which culture influences security behaviors, including management commitment signaling, peer norm establishment, and security operations integration. These findings specify culture’s often-abstract influence on behavior, providing actionable insights for culture transformation initiatives.

#### 6.1.2. Empirical contributions.

Empirically, this research provides rich documentation of employees’ perspectives on information safeguarding in an authentic organizational context, addressing the scarcity of qualitative employee-voice research in information security scholarship. The findings reveal nuanced understandings of how employees actually experience security imperatives, navigate competing demands, appraise their capabilities, and interpret organizational culture—insights that are inadequately captured by conventional survey methodologies.

The research illuminates the persistent knowing-doing gap, whereby employees possess substantial threat awareness yet exhibit inconsistent protective behaviors, and documents specific cognitive, contextual, and systemic factors that explain this paradox. By revealing variations in self-efficacy across employee segments, the research identifies specific population vulnerabilities that require targeted interventions. The documentation of peer influence mechanisms, management commitment signals, and security-usability tensions provides empirical grounding for understanding barriers to security effectiveness.

#### 6.1.3. Methodological contributions.

Methodologically, this research demonstrates the value of dual-source qualitative approaches combining naturalistic documentary data with semi-structured interviews. The analysis of 87 spontaneous employee comments extracted from internal systems provided authentic, unfiltered perspectives captured in natural organizational discourse, offering ecological validity often absent in researcher-prompted data. Triangulation with in-depth interviews enabled exploration of themes emerging from documentary analysis while accommodating participant elaboration on complex experiences. This methodological approach represents an innovative contribution to information security research, suggesting valuable strategies for future studies seeking to capture authentic employee perspectives.

The systematic thematic analysis process employed demonstrates rigorous qualitative methodology applicable to organizational information security contexts. By explicitly integrating theoretical frameworks (PMT) with inductive theme development, the research illustrates how theory-driven qualitative analysis can generate theoretically grounded yet empirically rich findings. The detailed documentation of coding procedures, theme development processes, and trustworthiness strategies provides methodological guidance for scholars conducting similar inquiries.

#### 6.1.4. Contextual contributions.

This research makes important contextual contributions by illuminating information security in Saudi Arabian organizational settings and the energy sector—both of which are dramatically underrepresented in international scholarship. The findings reveal how Saudi Arabia’s unique position as a global energy leader fosters a distinctive security consciousness, with employees recognizing national-security dimensions that transcend typical corporate concerns. The documentation of cultural factors, including collectivist orientation, hierarchical respect norms, and national security consciousness, provides valuable insights into information security in Middle Eastern contexts.

The energy sector focus addresses a critical gap in information security research, as this sector has been insufficiently examined despite its strategic importance. The findings illuminate sector-specific challenges, including the management of highly sensitive proprietary information, the presence of persistent state-sponsored threat actors, and the elevated consequences of security breaches. These contextual insights inform security practices across energy organizations globally and highlight how sector characteristics shape employee perspectives and behaviors.

#### 6.1.5. Practical implications.

For practitioners, this research offers multiple actionable implications for enhancing organizational information security effectiveness:

1. ***Cultivate Comprehensive Security Culture:*** Within this organizational context, and potentially in similar high-stakes critical infrastructure settings, organizations may benefit from moving beyond technical controls and policy pronouncements to cultivate authentic security cultures characterized by visible management commitment, supportive peer norms, and integration of security into daily operations. Leaders should personally model security behaviors, consistently acknowledge security considerations in decision-making, and address violations, regardless of the perpetrator’s status, to establish credibility.
2. ***Address Self-Efficacy Gaps Through Targeted Training:*** In this context, organizations may consider identifying employee segments with low self-efficacy and providing tailored training addressing their specific knowledge gaps and anxiety. Training should emphasize practical, scenario-based guidance relevant to actual work situations rather than generic presentations. Cultivating distributed security expertise and encouraging peer-to-peer learning can complement formal programs.
3. ***Reduce Response Costs Through User-Friendly Design:*** In high-stakes organizational settings such as this, security measures may benefit from minimizing workflow disruption through automated protections, intuitive interfaces, and streamlined procedures. Organizations should conduct usability testing of security requirements from an end-user perspective and redesign cumbersome processes that prompt workarounds. Making secure behaviors the path of least resistance enhances sustainable compliance.
4. ***Implement Continuous Communication and Learning:*** Organizations in similar critical infrastructure contexts should consider establishing ongoing security awareness initiatives, maintaining elevated consciousness over time, rather than relying on episodic campaigns following incidents. Systematic sharing of incident analyses, emerging threats, and effective practices enhances collective organizational intelligence. Creating mechanisms for employees to report concerns and receive guidance supports continuous learning.
5. ***Balance Technical and Human Approaches:*** Organizations operating in critical infrastructure sectors should consider investing in both technical controls and human-centered interventions, recognizing these as complementary rather than alternative approaches. Technical systems should compensate for human cognitive limitations through automation while preserving human judgment for contextual decisions. Human development initiatives should enhance threat awareness, self-efficacy, and response efficacy.
6. ***Establish Clear Accountability with Consistent Enforcement:*** In this context, organizations may benefit from clarifying individual security responsibilities, establishing transparent accountability mechanisms, and consistently enforcing consequences for violations. However, accountability systems should balance deterrence with positive reinforcement, recognizing and rewarding exemplary security behaviors to cultivate motivation beyond mere avoidance of compliance.

### 6.2. Limitations

This research acknowledges several limitations requiring consideration. First, the single-case study design within one Saudi Arabian energy corporation limits direct generalizability to other organizational contexts, sectors, or national settings. While the findings provide rich insights and theoretical propositions applicable beyond the specific case, empirical verification across diverse contexts remains necessary.

Second, the documentary data, while offering authentic employee voices, were not originally generated for research purposes and may reflect particular organizational circumstances surrounding their collection.

Regarding data availability: the raw dataset cannot be deposited in a public repository due to the sensitive and confidential nature of the organizational data (reflecting real security incidents and internal operational practices), the formal anonymity commitments made to participants, and the host organization’s confidentiality requirements under which data access was granted. This situation has been disclosed to PLOS ONE, and an exemption request has been submitted in accordance with the journal’s data policy. To support transparency within these constraints, an anonymized illustrative codebook with representative coded excerpts and the full coding framework has been prepared as S1 File. Additional data access may be granted to qualified researchers under a controlled data-sharing agreement ensuring confidentiality. So that access is not controlled by the author, and consistent with the Data Availability Statement and with the response entered in the PLOS ONE submission system, all such requests should be directed to the Research Ethics Committee at King Khalid University (ecm@kku.edu.sa), which is independent of the author and reviews requests in accordance with applicable ethical, legal, and confidentiality requirements. The comments were solicited following a major cyber incident, potentially amplifying security consciousness beyond typical baseline levels. Longitudinal research examining the evolution of perspectives over extended periods would provide valuable complementary insights.

Third, the cross-sectional design captures employee perspectives at a specific point in time, precluding examination of behavioral change processes or intervention effectiveness over time. Future longitudinal research tracking employees’ security perceptions and behaviors across multiple time points would illuminate dynamic change processes.

Fourth, while the dual-method qualitative approach provides rich insights, it relies on self-reported perspectives and experiences. Direct behavioral observation, system log analysis, or experimental manipulations could complement self-report data to more comprehensively triangulate behavioral patterns.

Fifth, the research focused primarily on employee perspectives, with limited examination of managerial or organizational system perspectives. Future research integrating multiple stakeholder viewpoints would provide a more comprehensive understanding of organizational security dynamics.

### 6.3. Future research directions

Several promising directions for future research emerge from this study:

1. ***Cross-Cultural Comparative Studies:*** Future research should examine information security perspectives across diverse national and organizational cultures, systematically comparing how cultural values shape threat perceptions, responsibility attribution, and protective behaviors. Comparative analysis of individualist and collectivist cultures, different national security contexts, and varied sectoral settings would illuminate the cultural boundary conditions of the findings.
2. ***Longitudinal Behavioral Change Research:*** Longitudinal studies tracking employees’ security perceptions, self-efficacy development, and behavioral patterns over extended periods would illuminate change processes and factors influencing sustained behavioral modification. Particularly valuable would be research examining how security consciousness evolves following organizational incidents and whether elevated awareness persists or decays over time.
3. ***Intervention Effectiveness Evaluation:*** Experimental or quasi-experimental research testing specific interventions suggested by this study—targeted training programs, automated security tools, culture transformation initiatives—would provide empirical evidence regarding intervention effectiveness. Rigorous evaluation designs that compare intervention and control conditions and measure actual behavioral outcomes (not merely attitudes or intentions) would generate actionable evidence for practitioners.
4. ***Technology Design Research:*** Future research should examine optimal security technology design from user experience perspectives, systematically testing interfaces, authentication mechanisms, and automated protections to identify configurations minimizing response costs while maximizing protection effectiveness. Participatory design approaches involving end-users in security technology development could yield innovations better aligned with user needs.

### 6.4. Conclusion

This study set out to understand how employees within a large multinational energy corporation in Saudi Arabia perceive, experience, and respond to information-safeguarding imperatives. It makes its central theoretical contribution by extending Protection Motivation Theory (PMT) beyond its original individual-level, health-behavior origins into an organizational, critical-infrastructure setting. The findings show that PMT’s core threat- and coping-appraisal constructs remain explanatorily useful in this context, but that their operation is conditioned by organizational moderators — namely management commitment, peer norms, and system usability — that are not specified in the original theory. This is the study’s key extension: it re-positions PMT as a sociotechnical rather than purely individual model, in which the well-documented knowing-doing gap (high threat awareness co-existing with inconsistent protective behavior) is explained not by deficient risk perception but by uneven self-efficacy and response-cost barriers that are themselves shaped by organizational culture. Applied for the first time to a Saudi Arabian energy-sector organization, this contextual application also demonstrates how collectivist orientation, hierarchical respect norms, and heightened national-security consciousness recalibrate the weight that employees place on coping appraisal relative to threat appraisal, a boundary condition that prior PMT research conducted in Western, non-critical-infrastructure settings could not reveal. Taken together, these findings suggest that PMT-informed security interventions in comparable high-stakes organizational environments are more likely to succeed when they target self-efficacy and response-cost barriers directly, rather than relying primarily on reinforcing threat awareness. While the single-case, exploratory design means these propositions should be treated as analytically generalizable rather than statistically representative, they offer a theoretically grounded starting point for future PMT research in organizational and cross-cultural information-security contexts.

## Supporting information

S1 File Anonymized codebook: Coding framework, code definitions and decision rules, representative coded excerpts, and inter-coder reliability documentation. (DOCX)
