# Supplementary material for: Employee perspectives on safeguarding information: An exploratory study in a multinational energy corporation
Source: PLoS One. 2026 Oct 7;21(10):e0349656. doi: 10.1371/journal.pone.0349656 (PMC13645051; doi:10.1371/journal.pone.0349656)
Supplement: Supplementary file 1 [file pone.0349656.s001.docx]

**S1 File**

**Anonymized Codebook: Coding Framework and Representative Excerpts**

Manuscript: Employee Perspectives on Safeguarding Information: An Exploratory Study in a Multinational Energy Corporation

Manuscript ID: PONE-D-26-09284 | PLOS ONE

Corresponding Author: Dr. Mohamed Mohiya | ORCID: 0000-0002-6701-3071

**1. Purpose and Scope of This Document**

This codebook was developed to support methodological transparency in accordance with PLOS ONE data policy. It provides: (1) the full coding framework used in the thematic analysis, including code definitions and decision rules; (2) anonymized representative excerpts illustrating each code; and (3) documentation of the inter-coder reliability procedure.

The raw dataset — comprising 87 employee documentary comments and 24 semi-structured interview transcripts — cannot be deposited in a public repository due to the sensitive and confidential nature of the organizational security data, formal anonymity commitments to participants, and the host organization’s confidentiality requirements. All excerpts included in this document have been fully anonymized: participant names, job titles, department identifiers, specific dates, and any other potentially identifying information have been removed or replaced with generic descriptors.

Additional data access may be granted to qualified researchers under a controlled data-sharing agreement ensuring confidentiality; requests should be directed to the Research Ethics Committee at King Khalid University (ecm@kku.edu.sa), which is independent of the author and reviews requests in accordance with applicable ethical, legal, and confidentiality requirements.

**2. Data Sources**

**2.1 Documentary Data (Source A)**

Eighty-seven (87) employee comments were extracted from the organization’s internal electronic communication system. These comments were submitted in response to an internal initiative encouraging employee input on information-safeguarding practices, launched after a significant cyber incident the organization experienced. Because the internal system permitted only one submission per employee account, the 87 comments correspond to 87 distinct employees; individual identities were not retained, to preserve anonymity. In this codebook, documentary excerpts are identified as [Doc-xx] where xx is an anonymized comment number.

**2.2 Interview Data (Source B)**

Twenty-four (24) semi-structured interviews were conducted with purposively selected employees across five functional departments (operations, engineering, IT/cybersecurity, administration, finance). Interviews lasted 45–60 minutes. Nine interviews were conducted in Arabic and translated into English via a professional bilingual translator with back-translation verification. In this codebook, interview excerpts are identified as [Int-xx, Role] where the role descriptor is a generalized functional category (e.g., ‘Senior Manager’, ‘IT Professional’, ‘Operations Staff’) rather than a specific job title, to preserve anonymity.

**3. Analytical Approach**

Thematic analysis followed Braun and Clarke’s (2006) six-phase reflexive framework, combining inductive coding (allowing themes to emerge from the data) with deductive coding informed by constructs of Protection Motivation Theory (PMT). Analysis was managed using NVivo qualitative data analysis software. The coding process proceeded through: (1) data familiarization; (2) initial code generation; (3) theme construction; (4) theme review; (5) theme definition and naming; and (6) synthesis and write-up.

A second independent researcher with expertise in qualitative organizational research coded a randomly selected 25% sub-sample (approximately 22 documentary comments and 6 interview transcripts) using this same coding protocol. Cohen’s Kappa was κ = 0.82, indicating strong agreement (Landis & Koch, 1977). Disagreements (18% of initially coded segments) were resolved through discussion and consensus, with code definitions refined accordingly.

**4. Full Coding Framework with Definitions and Anchor Examples**

The table below presents all codes organized by theme and sub-theme. All excerpts are anonymized.

**Theme 1: Heightened Threat Awareness and Perceived Vulnerability**

PMT Dimension: Threat Appraisal (Perceived Severity + Perceived Vulnerability).

Definition: Segments in which participants articulate awareness of information security threats, express perceived vulnerability to those threats, or describe the severity of potential consequences for the organization or themselves.

| **Code Name** | **Definition** | **Anchor Example (anonymized)** | **Source** |
| --- | --- | --- | --- |
| 1.1: Cyber Threat Recognition | Participant identifies specific cyber threats (hacking, phishing, viruses, data breaches) and acknowledges their real and present danger. | "Every time hackers are more aggressive... we, everybody, must be suspicious about anything that may be a potential exposure." [Doc-07] | Documentary |
| 1.2: Organizational Vulnerability Acknowledgment | Participant acknowledges that the organization is or has been vulnerable to security incidents, citing past incidents or current gaps. | "Lessons learned as a result of the cyber-attack are not communicated openly... the big picture of protecting company information is not clear to all employees." [Doc-13] | Documentary |
| 1.3: Perceived Severity of Consequences | Participant articulates serious potential consequences of security breaches for the organization, its assets, or national security. | "Information is a very critical enterprise asset. Without information, there is no such thing as a wise decision." [Doc-44] | Documentary |
| 1.4: National Security Dimension | Participant frames information explicitly within the context of national security or critical infrastructure. | "Upstream information items are a national asset. The risk of exposure varies from one information asset to another. Safeguarding this information... requires an ongoing and collaborative effort." [Doc-87] | Documentary |
| 1.5: Post-Incident Heightened Awareness | Participant cites a specific past cyber incident as a catalyst for heightened security awareness. | "Previous experience of [the organization] taught us to be vigilant and meticulous in safeguarding intellectual assets." [Doc-16] | Documentary |
| 1.6: Third-Party and Insider Risk Awareness | Participant identifies risks from external partners, contractors, or insider access as distinct threat vectors. | "Business partners are a weak link... go to a vendor site and look for a list of their achievements. If [the company] is one of their customers, they’re likely to point this out." [Doc-46] | Documentary |

**Theme 2: Individual Responsibility, Accountability, and Role Ambiguity**

PMT Dimension: Coping Appraisal (Response Efficacy + Self-Efficacy) intersecting with Role Clarity.

Definition: Segments addressing the distribution of responsibility for information security between individuals and the organization, including expressions of personal accountability, boundary ambiguity, and resistance or skepticism.

| **Code Name** | **Definition** | **Anchor Example (anonymized)** | **Source** |
| --- | --- | --- | --- |
| 2.1: Individual Accountability Assertion | Participant explicitly accepts personal responsibility for safeguarding information. | "Safeguarding information is everyone’s responsibility. Everyone has a responsibility for safeguarding information." [Doc-21] | Documentary |
| 2.2: Organizational vs. Individual Boundary Ambiguity | Participant expresses uncertainty about where individual responsibility ends and organizational/IT responsibility begins. | "There is a big gap between end users and professionals who have the technical expertise to set safeguards. Professionals should think from the end user’s non-technical mindset." [Doc-24] | Documentary |
| 2.3: Skepticism Toward Security Measures | Participant questions the effectiveness, practicality, or design of organizational security policies or procedures. | "One year down the line, I feel we have to draw a moderate line between usability and security. Too many internet restriction negatively impacts smooth operations." [Doc-83] | Documentary |
| 2.4: Compliance Cost Awareness | Participant acknowledges tension between security compliance and operational efficiency or workflow convenience. | "Sadly, with the new procedures in protecting our privacy and information, a lot of good websites — that are work-related — are banned, and personal emails are blocked." [Doc-28] | Documentary |
| 2.5: Self-Efficacy Variation | Participant expresses uncertainty about their own ability to perform security behaviors or identifies knowledge gaps. | "It is not correct to say that everybody is capable of backing up their data. Most employees are experts in their respective fields, but might not be aware of the IT activities they need to perform." [Doc-72] | Documentary |

**Theme 3: Organizational Security Culture and Management Commitment**

PMT Dimension: Contextual Moderator of both Threat and Coping Appraisal.

Definition: Segments addressing the organizational security culture, management leadership in security, peer norms, and the role of organizational values in shaping security behavior.

| **Code Name** | **Definition** | **Anchor Example (anonymized)** | **Source** |
| --- | --- | --- | --- |
| 3.1: Management Leadership in Security | Participant identifies management commitment, championship, or modeling as critical to security culture. | "Management championship shall help to drive end-user adoption. Information governance needs to be formalized at the corporate level." [Doc-45] | Documentary |
| 3.2: Collective/Cultural Security Norms | Participant references shared organizational values, culture, or collective identity as a security enabler. | "Our [organizational] culture protects us even in unconscious behavior." [Doc-14] | Documentary |
| 3.3: Peer Accountability and Social Influence | Participant notes the role of peer behavior, social norms, or collective responsibility in shaping individual security actions. | "We have to promote a well-organized campaign to have every user accountable of his/her act." [Doc-13] | Documentary |
| 3.4: Cross-Departmental Coordination | Participant highlights the need for cross-departmental collaboration or between IT and business units to ensure effective security. | "IT cannot work alone! We would like you to be more involved with us, speak to us. It is your data after all." [Doc-82] | Documentary |
| 3.5: Organizational Values and Integrity | Participant explicitly links information security to broader organizational values such as integrity, loyalty, and ethical behavior. | "Company culture values integrity. A formalized Statement of Integrity spells out our responsibilities to act ethically. Confidentiality is an integral part of integrity." [Doc-31] | Documentary |

**Theme 4: Technical Controls versus Human-Centered Factors**

PMT Dimension: Response Efficacy and Response Cost.

Definition: Segments addressing the relative role of technical solutions versus human behavior and judgment in information protection, including evaluations of technical measure effectiveness and usability trade-offs.

| **Code Name** | **Definition** | **Anchor Example (anonymized)** | **Source** |
| --- | --- | --- | --- |
| 4.1: Technical Solutions Advocacy | Participant recommends or endorses specific technical controls as effective safeguards. | "Effective IT computing resources, security, and network traffic monitoring are the best ways to ensure that information in transit or stored does not get leaked." [Doc-06] | Documentary |
| 4.2: Human Factor as Dominant Risk | Participant identifies human behavior, error, or negligence as the primary security vulnerability. | "Regardless of how much IT puts in protection systems and measures, WE, the human users, remain the weakest link in the chain!" [Doc-83] | Documentary |
| 4.3: Usability-Security Trade-off | Participant identifies tension between security measures and operational usability or workflow efficiency. | "Having too many/complicated rules can backfire with more problems than what we started with." [Doc-24] | Documentary |
| 4.4: Integrated Technical-Human Approach | Participant advocates for solutions that combine technical controls with human-centered training or behavioral change. | "Digital security has become an imperative. While antivirus programs can help defend against known problems, they aren’t much use against new or inventive assaults." [Doc-25] | Documentary |
| 4.5: Physical Security Dimension | Participant extends security concerns to physical or hard-copy information management. | "Printed records are usually overlooked in protection. Information thieves are not just on the web — they are also looking for information in hard copy." [Doc-07] | Documentary |

**Theme 5: Knowledge Gaps, Training Needs, and Continuous Learning**

PMT Dimension: Self-Efficacy Enhancement through Education.

Definition: Segments addressing gaps in security knowledge, training insufficiency, recommendations for awareness programs, and the importance of continuous learning for security behavior.

| **Code Name** | **Definition** | **Anchor Example (anonymized)** | **Source** |
| --- | --- | --- | --- |
| 5.1: Security Knowledge Gap Identification | Participant identifies a specific gap in their own or colleagues’ security knowledge. | "Many people did not experience the cyber-attack last year. So for all the newbies... back up your work on the network." [Doc-05] | Documentary |
| 5.2: Training and Awareness Program Recommendation | Participant advocates for formal training, awareness campaigns, or educational programs. | "Continuous educational sessions and programs on safeguarding information are required to promote the awareness level of all employees, especially newcomers." [Doc-80] | Documentary |
| 5.3: Early Education and Socialization | Participant recommends embedding security education from early stages (onboarding, schooling, family). | "Relevant education and awareness should start in schools to produce a future cyber-literate society." [Doc-04] | Documentary |
| 5.4: Experiential Learning from Incidents | Participant frames past incidents as learning opportunities and advocates for the systematic capture and sharing of lessons learned. | "It is good that this topic is addressed one year after the cyber-attack. Lessons learned include that each operational organization should have a set of internal guidelines." [Doc-85] | Documentary |
| 5.5: Personal Security Commitment and Peer Education | Participant expresses personal motivation to improve security behavior and to educate colleagues. | "I firmly believe that once a person truly ‘feels’ the importance of cyber security, they’ll take personal interest in it. I suggest anyone who wants to contribute conduct an awareness session within their organization." [Doc-81] | Documentary |

**5. Coding Decision Rules**

The following decision rules were applied consistently throughout the coding process:

Rule 1 — Unit of analysis: The unit of coding was the semantic unit (a phrase, sentence, or passage conveying a coherent meaning related to a code definition), not the individual word or the full paragraph.

Rule 2 — Multiple coding: A single semantic unit could be assigned to more than one code where it addressed multiple dimensions simultaneously. This accounts for the fact that percentages in Table 4 of the manuscript sum to more than 100%.

Rule 3 — Deductive-inductive balance: PMT constructs provided deductive scaffolding, but codes were permitted to emerge inductively where participant data introduced dimensions not anticipated by PMT (e.g., national security framing, physical security concerns).

Rule 4 — Disconfirming evidence: Segments expressing skepticism, resistance, or critique of security measures were actively coded (not excluded) and are represented in Code 2.3 (Skepticism Toward Security Measures).

Rule 5 — Anonymization standard: Any excerpt included in publications or supplementary materials had all identifying information removed or replaced with a generic descriptor before inclusion.

**6. Inter-Coder Reliability Documentation**

| **Code Name** | **Definition** | **Anchor Example (anonymized)** | **Source** |
| --- | --- | --- | --- |
| Sub-sample size | 25% of the full dataset | ≈22 documentary comments + 6 interview transcripts |  |
| Second coder profile | Independent researcher with qualitative organizational research expertise | Blind to the primary researcher’s codes during the independent coding phase |  |
| Agreement metric | Cohen’s Kappa (κ) | Landis & Koch (1977) benchmark: κ ≥ 0.80 = strong agreement |  |
| Kappa coefficient | κ = 0.82 | Indicates strong inter-coder agreement |  |
| Initial disagreement rate | 18% of coded segments | Resolved through structured discussion and consensus |  |
| Resolution procedure | Discussion and consensus | Coding framework refined following resolution; final codes reflect consensus decisions |  |

**7. References**

Braun, V., & Clarke, V. (2006). Using thematic analysis in psychology. Qualitative Research in Psychology, 3(2), 77–101.

Landis, J. R., & Koch, G. G. (1977). The measurement of observer agreement for categorical data. Biometrics, 33(1), 159–174.

Rogers, R. W. (1975). A protection motivation theory of fear appeals and attitude change. Journal of Psychology, 91(1), 93–114.

End of S1 File

All raw data excerpts are anonymized. No identifying information is disclosed.
